# The efficacy of Rhodiola Rosea based on DTI image Segmentation Algorithm for patients with delayed Encephalopathy caused by CO poisoning

**DOI:** 10.12669/pjms.37.6-WIT.4830

**Published:** 2021

**Authors:** Yuming Gao, Haitao Cui, Wei Ren, Bing Han

**Affiliations:** 1 Yuming Gao, Bachelor’s Degrees. Department of Emergency, Jiaozhou Central Hospital of Qingdao, QingDao, 266300, Shandong, China; 2 Haitao Cui, Bachelor’s Degrees. Clinical Laboratory, Jiaozhou Central Hospital of Qingdao, QingDao, 266300, Shandong, China; 3 Wei Ren, Bachelor’s Degrees. Intensive Care Unit, Jiaozhou Central Hospital of Qingdao, QingDao, 266300, Shandong, China; 4 Bing Han, Bachelor’s Degrees. Department of Emergency, Jiaozhou Central Hospital of Qingdao, QingDao, 266300, Shandong, China

**Keywords:** Rhodiola: image segmentation algorithm, DTI, nitric oxide synthase, CT, delayed encephalopathy

## Abstract

**Objectives:**

By using DTI image segmentation algorithm investigate the effect of large plants Rhodiola injection on myocardial injury in patients with acute severe CO poisoning (ACOP), and to explore the clinical and CT delayed encephalopathy after ACOP.

**Methods:**

Seventy-two ACOP patients were randomly divided into control and observation group, 36 cases in each group from December 2015 – December 2017. The control group received hyperbaric oxygen, mannitol, dexamethasone, citicoline injection, gangliosides, dracone; observation group were large strain Rhodiola injection treatment group based on the once daily for two weeks of continuous treatment. The head CT, head MRI results were analyzed retrospectively.

**Results:**

(1) hsCRP and ET-1 in the observation group were significantly lower than those in the control group, and VEGF was significantly higher than that in the control group (P<0.01). No, NOS, and iNOS were significantly lower than those of the control group (P<0.01); (2) CT images of 16 cases showed bilateral symmetrical fusion lesions with blurred edges, low density, and oval center around the ventricle; (3) MRI showed that the lesion was located in the cerebral cortex, white matter lateral ventricle and/or basal ganglia in 12 cases.

**Conclusion:**

Rhodiola can reduce myocardial vascular endothelial cell injury, improve cardiac function, and protect the damaged myocardium. Meanwhile, after acute CO poisoning delayed encephalopathy early for CT and MRI examination facilitate analysis and prognosis of the disease.

## INTRODUCTION

Due to high morbidity, mortality and poor prognosis, acute severe CO poisoning (ACOP) has become a hot topic in the medical community.^1^ Hypoxia after CO poisoning is mostly manifested as myocardial dysfunction, arrhythmia, myocardial infarction.^2^ Rhodiola rosea can slow the rate of myocardial oxygen consumption, expand the coronary arteries, increase blood flow in the coronary arteries, reduce peripheral resistance.^3^ Our hospital treats patients with acute and severe CO poisoning with Rhodiola rosea to investigate the effects on myocardial function, vascular inflammation factors, vascular endothelial growth factor (VEGF) and NO.

Delayed encephalopathy after acute carbon monoxide poisoning (DEACMP) after CO poisoning is easy to be ignored and cause serious consequences. Skull CT MRI can detect brain lesions after CO poisoning early.^4^-^5^ The data of 72 DEACMP patients in our hospital are summarized as follows.

## METHODS

(1) Graph cutting in discrete scalar space. The graphic cutting method is an interactive image segmentation method. First, the designer selects some pixels as the target object and background. The category of the remaining pixels is unknown. Then the pixels are assigned as vertices. Finally, the minimum cut method of the graph is to segment the image into target objects and background two parts.

Suppose graph *G≤V,E>V* is a set of nodes, E is a set of connection relations between pixels and neighborhoods, and the weight of edges is a function of the degree of similarity between two pixel-specific attributes. Eight neighborhood connections are selected. Image segmentation is a binary classification of nodes of unknown class in V. For the convenience of graph construction and segmentation calculation, two dummy points are added: the sending point and the receiving point (as shown in *s,t* in Fig.1, the other nodes are pixels, and *ω* is the edge weight). Construct the objective function:


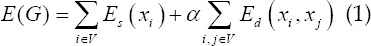


In the formula: *x_i_,y_i_* is the generic of node *^i,j^*; *E_s_(x_i_)* is the penalty of node i belongs to *x_i_*; *E_d_(x_i_,x_j_)* is the penalty of node 0<*α*<1 belongs to *x_i_,y_i_* respectively; α is the expansion factor, *^i,j^*. Assume that *D_i_^o^* , *D_i_^B^* is the distance from node i to the target and background set, B, O, and U are the background, target, and unknown pixel sets, respectively, and *P_i_,P_j_* is the segmentation attribute of *i,j* nodes. Then, *E_s_(x_i_)*, *E_d_(x_i_,x_j_)* is defined as:


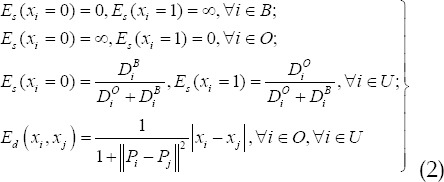


The first two penalties ensure that the target seed is segmented into the target set, and the background seed is segmented into the background set; the third penalty guarantees that general pixels are segmented into the closest set; the fourth term is a penalty for the segmentation boundary of the target set. Finally, the maximum flow algorithm is used to minimize to segment the target area.

Graphic cutting of discrete tensor space. It needs to be extended in the following two aspects: First, the image element’s segmentation characteristic standard is mathematically extended from a scalar to a tensor. Define the similarity between two diffusion tensors:


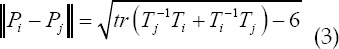


Equation (3) shows that the larger the difference between *T_i_, T_j_* and tensor, the greater the similarity between the diffusion characteristics between them, which is ∞ in the limit case; otherwise, the smaller the difference between the diffusion characteristics, the smaller the distance, and the distance *T_i_=T_j_*, in the limit case. Equal to 0. Starting from the definition of diffusion tensor similarity, the effective distance between the unknown generic node i to the target set and the background set is defined as the average value of the similarity between node i and each set:


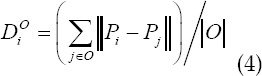



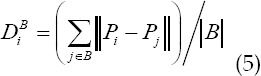


In the formula: *B* is the number of elements in the background set, and *O* is the number of elements in the segmentation target set. The maximum flow algorithm is used to minimize *E(G)*, and the carcass segmentation region is obtained.

Research objects. 72 ACOP patients were randomly divided into control and observation group, 36 cases in each group. The patients in December 2015 – December 2016 admitted to the control group; patients in January 2017 – December 2017 admitted to the observation group. 20 males and 16 females in the control group; age 38~65 years (49.3±5.6) years; coma is 2~10h (6.3±2.5)h; coronary heart disease 12 cases of cerebral infarction in three cases, seven cases of hypertension, diabetes eight cases. Observation group 19 males and 17 females; mean age was (50.2±5.5) years old, coma time is 2~11h (5.9±2.7)h, 11 cases with coronary heart disease, cerebral infarction in four cases, eight cases of hypertension, diabetes seven cases. General information on two groups of patients, the difference was not statistically significant (P>0.05). Both in the acute phase of coma, coma time 5h~9d. 16 cases of coma delayed encephalopathy clear from 4 hours to 30 days, an average of 17 days. There are eight cases of mental symptoms, incontinence five cases; grand mal seizures in two cases. four routine lumbar punctures, cerebrospinal fluid pressure, conventional, biochemical, immunological and cytological examination were normal. EEG 18 cases were treated: 15 cases of exception, where extensive severe abnormalities nine; five cases of moderately abnormal widely; broad mild abnormalities in one case. EEG abnormalities primarily for a wide range of irregular diffuse slow, continuous long-range, seriously appear flat waveform, individual performance spikes, sharp wave or spike and wave. 618 cases underwent head CT. 12 cases received routine MRI examination.

Both met the GBZ 23-2002 “Diagnostic Criteria for Occupational Acute Carbon Monoxide Poisoning” [7]. The patients were all severely poisoned. All patients volunteered to participate in this experiment. Exclusion criteria: severe liver dysfunction, renal dysfunction, heart valve disease, meningitis, hematological disease, malignant tumor and other diseases.

After admission, the control group was treated with hyperbaric oxygen, while mannitol, dexamethasone, citicoline injection, and ganglioside (produced by Qilu Pharmaceutical Company, production batch number H20056783) were given intramuscularly, dracone (Produced by Kunming Jida Pharmaceutical Company, batch number H20080495) Intravenous drip. The observation group was given 10 ml of Rhodiola rosea injection (produced by Tonghua Yusheng Pharmaceutical Co., Ltd., Z20110704) based on the control group, 250 ml of saline was added intravenously, once daily and the treatment was continued for 2 weeks. Patients in the two groups received the same drug lot number.

Observation indicators. (1) fasting blood samples were collected, automatic biochemical analyzer serum CK-MB activity; troponin (of cTnI), myoglobin (Myo) Content. Using color Doppler ultrasound cardiac LVEF detecting a patient. (2) respectively detecting a hsCRP and VEGF levels using radioimmunoassay of endothelin (ET-1) was detected by the ELISA.^8^ (3) With reference to kit, chemical colorimetric detection of NO content; NOS and iNOS activity.

(5) Statistical processing. SPSS 17.0 statistical analysis was used. Count data comparison was performed by χ^2^ test. Measurement data were expressed by *x̅±s*. Two groups were compared by t test. Multiple groups were compared by analysis of variance. P<0.05 was considered statistically significant.

## RESULTS

CT results. Head CT examination showed no abnormality. The skull CT examination showed delayed encephalopathy 1 to 20 days after acute CO poisoning. The lesions were mostly located in the white matter of the brain, and the basal ganglia area mainly involved the pale bulbs.

Ten cases of pure white matter involving the paraventricular or hemisphere center of the hemisphere side of the brain were involved, and five cases of basal ganglia or pale bulbous lesions were involved in the hemisphere side of the cerebral ventricle and hemisphere center, and 1 case had cerebral cortex damage.

The lesions were basically symmetrically distributed, and the lesions ranged in size and divergent. The morphology was spot-shaped, spot-shaped, some melted into clumps, the edges were slightly fuzzy, and there was little strengthening, and no placeholder effect. five cases were only involved in the paraventricular or hemisphere center white matter of the cerebral hemisphere side. Involving the paraventricular side of the hemisphere side of the brain and the center of the hemi-ovum, 6 cases with basal ganglia or pale bulbous lesions, and one case with damaged cerebral cortex. The lesion morphology was basically consistent with the CT findings. It shows low signal or equal signal at T1, and high signal at T2 and T2flair. Some patients showed that the gray matter interface of brain tissue was unclear on T1-weighted images.

All patients underwent hyperbaric oxygen therapy, 1 hour a day, 10 days as a course of treatment, given 2 to 4 courses or until the condition improved. In the early onset of delayed encephalopathy after acute CO poisoning, dexamethasone was administered intravenously at a dose of 10-20 mg daily, and it was gradually reduced to discontinuation after 5-7 days. After the above treatment, the clinical symptoms of all patients were improved.Heart function comparison. CKMB, cTnI, Myo were significantly lower than the control group, LVEF was significantly higher (P<0.01). Table-I.

**Table-I:** Comparison of cardiac function (*x̅*±*s*, n = 36).

*Group*		*Observation group*	*Control group*	*t*	*P*
CK-MB(U/L)	Before	35.12±5.36	34.98±5.17	0.113	>0.05
	After	9.43±2.25a	16.07±2.87a	10.924	<0.05
cTnI(ng/ml)	Before	0.14±0.08	0.15±0.11	0.441	>0.05
	After	0.25±0.12a	0.43±0.13a	6.105	<0.05
Myo(μg/L)	Before	158.74±44.72	156.83±43.57	0.184	>0.05
	After	34.25±11.02a	40.36±11.78a	2.273	<0.05
LVEF (%)	Before	42.76±5.08	43.15±4.86	0.333	>0.05
	After	60.32±4.56a	52.53±4.86a	7.013	<0.05

*Note:* After t test, compared with the same group before treatment, aP<0.01. CK-MB-creatine kinase isoenzyme; cTnI-troponin; Myo-myoglobin; LVEF-left ventricular ejection fraction.

Comparison of inflammatory factors and vascular endothelial function. HsCRP and ET-1 were significantly lower than the control group, of VEGF significantly higher (P<0.01). See Table-II.

**Table-II:** Comparison of inflammatory factors and vascular endothelial function (*x̅*±*s*, n = 36).

*Group*		*Observation group*	*Control group*	*t value*	*P value*
hsCRP(mg/L)	Before	3.76±1.05	3.98±1.37	0.765	>0.05
	After	2.13±0.86a	3.25±0.89a	5.429	<0.05
VEGF (ng/L)	Before	60.49±10.56	59.87±10.23	0.253	>0.05
	After	75.18±10.87a	68.75±10.46a	2.557	<0.05
ET-1(μmol/L)	Before	85.58±19.25	86.93±18.78	0.301	>0.05
	After	42.36±16.49a	58.42±15.93a	4.203	<0.05

*Note:* t-test, compared with the same group before treatment, aP<0.01. hsCRP- high sensitivity C-reactive protein; VEGF-vascular endothelial growth factor; ET-1- endothelin.

NO, NOS, iNOS were significantly lower than the control group, the differences were statistically significant (P<0.01). See Table-III.

**Table-III:** Comparison of NO, NOS, and iNOS in serum (*x̅*±*s*, n = 36).

*Group*		*Observation group*	*Control group*	*t*	*P*
NO(μmol/L)	Before	63.47±10.86	62.98±12.52	0.177	>0.05
	After	52.13±8.89	57.46±9.25	2.493	<0.05
NOS(U/L)	Before	0.032±0.007	0.031±0.008	0.564	>0.05
	After	0.017±0.006	0.022±0.005	3.841	<0.05
iNOS(U/L)	Before	0.020±0.006	0.019±0.005	0.768	>0.05
	After	0.012±0.005	0.017±0.004	4.685	<0.05

## DISCUSSION

The clinical manifestations of ACOP are mainly mental retardation, mental symptoms, tremor, dystonia, incontinence and paralysis.^9^ The EEG of delayed encephalopathy after ACOP mainly changed to irregular diffuse slow wave.^10^ The related pathogenesis has not been fully elucidated so far. Hypoxic-ischemic theory, cytotoxicity theory, and lipid peroxidation are the most common theories. In recent years, some authors have suggested that brain allergies are involved in the pathogenesis.^11^,^12^ The autopsy results revealed that the early pathological changes of CO poisoning were mainly gray matter lesions, and the most important changes in the brain were acute cerebral edema, capillary and venous dilation and hemorrhage and necrosis caused by hypoxia.

The imaging data found that: (1) the lesions mainly affected the white matter around the bilateral ventricles and the semi-oval center; (2) the symmetry of the basal ganglia area (mainly pale balls), or combined with the aforementioned white matter damage; (3) early gray matter interface is not clear, brain atrophy and other non-characteristic changes. Therefore, CT and MRI can better reflect the scope and severity of brain damage of delayed encephalopathy ACOP. In this group, clinical symptoms and electroencephalogram were improved to varying degrees after treatment with hyperbaric oxygen, hormones, and energy.

Animal experiments confirmed that Rhodiola rosea treatment of myocardial infarction rats increased rat VEGF, inhibited the production of inflammatory factors, improved blood viscosity, inhibited platelet aggregation, promoted angiogenesis, and improved blood circulation.^21^ Other studies have shown that Rhodiola rosea can increase high density lipoprotein, reduce low density lipoprotein, and regulate the effect of blood lipids.^15^-^17^

## CONCLUSIONS

Based on the above results, we can find that Rhodiola significantly improves cardiac function, reduces inflammatory factors, increases VEGF to repair damaged cells, inhibits the release of NO, thereby protecting myocardial cells. Rhodiola rosea injection can reduce inflammatory response, reduce myocardial vascular endothelial cell damage, promote new blood vessels, improve cardiac function, protect damaged myocardium. Patients after ACOP should be closely observed. Once symptoms occur, CT and MRI should be performed as soon as possible to detect and treat DEACMP early and help judge the prognosis.

### List of Abbreviations:

**ICD-10:** International Classification of Diseases-10.

**WHO:** World Health Organization.

**SDQ:** Strengths and difficulties questionnaire.

**CGI:** Clinical global Impression Scale.

**LOS:** length of stay.

**CAMHS:** Child & Adolescent Mental Health

Services.

**LMIC:** Low and Middle-Income countries.

### Authors Contribution:

**YG:** Conceived the study, literature review, participated in its design, coordination, analyzed the data and helped to draft the manuscript and also the responsible and accountable for the accuracy or integrity of the work

**HC & WR:** Helped in design, data collection, article drafting & critical revision.

**BH:** Takes the responsibility and is accountable for all aspects of the work in ensuring that questions related to the accuracy or integrity of any part of the work are appropriately investigated and resolved.
